# Impact of liberal preoperative clear fluid fasting regimens on the risk of pulmonary aspiration in children (EUROFAST): an international prospective cohort study

**DOI:** 10.1016/j.bja.2025.03.031

**Published:** 2025-05-26

**Authors:** Peter Frykholm, Ali-Reza Modiri, Anna Klaucane, Christiane E. Beck, Lionel Bouvet, Rebecca S. Isserman, Vimmi Oshan, Paul A. Stricker, Vinícius C. Quintão, Robert Frithiof, the EUROFAST Collaborative Group

**Affiliations:** 1 Department of Surgical Sciences, Section of Anaesthesiology and Intensive Care Medicine, Uppsala University, Uppsala, Sweden; 2 Centre for Paediatric Anaesthesia and Intensive Care Research, Uppsala University, Uppsala, Sweden; 3 Department of Anesthesiology and Intensive Care Medicine, Hannover Medical School, Hannover, Germany; 4 Department of Anesthesiology and Intensive Care, Hospices Civils de Lyon, Femme Mère Enfant Hospital, University of Lyon, Université Claude Bernard, Lyon, France; 5 Department of Anesthesiology and Critical Care Medicine, The Children's Hospital of Philadelphia and Perelman School of Medicine at the University of Pennsylvania, Philadelphia, PA, USA; 6 Department of Paediatric Anaesthesia, Royal Manchester Children's Hospital, Manchester University NHS Foundation Trust, Manchester, UK; 7 Instituto da Criança e do Adolescente, Hospital das Clínicas HCFMUSP, Faculdade de Medicina, Universidade de São Paulo, São Paulo, Brazil

**Keywords:** clear fluids, general anaesthesia, paediatric anaesthesia, preoperative fasting, pulmonary aspiration

## Abstract

**Background:**

Preoperative fasting regimens designed to minimise the risk of pulmonary aspiration have undergone significant changes, but unequivocal evidence of the safety of reducing clear fluid fasting has been lacking. We compared the risk of pulmonary aspiration in children using three different recommendations for clear fluid fasting.

**Methods:**

In this prospective multicentre cohort study, centres with >1000 paediatric anaesthesia cases per year were eligible. Regurgitation events, whether they were transient or led to consequences affecting postoperative care, were reported in detail. All centres also reported the number of anaesthetised children per year and which preoperative fasting regimen they used.

**Results:**

The 31 participating centres contributed a total of 306 900 anaesthetic procedures. The incidence of confirmed pulmonary aspiration was 1.18:10 000 in the sip-til-send group, 0.96:10 000 in the ≥1 h group, and 1.83:10 000 in the control group. There was no mortality as a result of aspiration. The 95% confidence intervals of the differences in confirmed pulmonary aspiration between the control group and the ≥1 h clear fluid fasting and the sip-til-send group were –0.344 to 3.76 and –1.48 to 3.63, respectively. Both sip-til-send and ≥1 h clear fluid fasting were statistically noninferior to ≥2 h clear fluid fasting regarding the incidence of confirmed aspiration, transient regurgitation, and regurgitation leading to escalation of care or intensive care.

**Conclusions:**

The study provides evidence for the safety of reducing preoperative fasting time for clear fluids in children aged <16 yr from 2 h to ≤1 h.

**Editor's key points**

• Preoperative fasting regimens have undergone significant changes, but unequivocal evidence of the safety of reducing clear fluid fasting has been lacking.
• This prospective multicentre cohort study compared the risk of pulmonary aspiration in children <16 yr of age using three different recommendations for clear fluid fasting.
• The sip-til-send and ≥1 h clear fluid fasting were noninferior to ≥2 h clear fluid fasting with regards to the primary outcome of confirmed aspiration, and secondary outcomes of regurgitation with symptoms and escalation of care.
• Reducing the minimum recommended preoperative fasting time for clear fluids from 2 h to ≤1 h is safe in children undergoing general anaesthesia.

Preoperative fasting regimens designed to minimise the risk of pulmonary aspiration have undergone significant changes during the last three decades.^1^ Previously, nil-by-mouth from midnight was considered necessary to prevent pulmonary aspiration during general anaesthesia, but this regimen has been replaced by more liberal recommendations.

Current fasting guidelines are designed to balance the risk of pulmonary aspiration and the harmful effects of prolonged preoperative fasting. However, there is increased awareness that many patients, both children and adults, suffer from prolonged fasting. This is in spite of the reasonable nominal limits of 6 h for solid food, 4 h for breast milk, and 2 h for clear fluids before induction of anaesthesia that has become global standard of care since the first ASA guideline was published in 1999.^2^ Prolonged fasting can be harmful, especially in children, and also in adults with poor nutritional status. The most recent guideline on preoperative fasting in children by the European Society of Anaesthesia and Intensive Care (ESAIC) recommends that children should be encouraged to drink clear fluids until 1 h before induction of anaesthesia, and acknowledges that even shorter fasting requirements for clear fluids are feasible.^3^ This recommendation was based on large quality improvement projects and observational studies that show that the risk of prolonged fasting is decreased when reducing the minimum clear fluid fasting time from 2 h to ≤1 h.^4^, ^5^, ^6^ However, to our knowledge no study has convincingly demonstrated that the shorter fasting time does not increase the risk of pulmonary aspiration. The problem from a methodological perspective is that confirmed pulmonary aspiration associated with general anaesthesia is uncommon such that a very large sample size (well above 100 000) is needed to prove similar safety.

In the EUROFAST study, we aimed to compare the risk of pulmonary aspiration between three different regimens for clear fluid fasting (sip-til-send, ≥1 h, and ≥2 h clear fluid fasting). We hypothesised that preoperative fasting regimens allowing clear fluids up to ≤1 h before elective surgery would not lead to an increased incidence of pulmonary aspiration when compared with the traditional 2-h rule (noninferiority).

## Methods

This was a prospective multicentre cohort study that began as a multicentre audit in Sweden, but was expanded to an international project to increase the sample size and generalisability. Ethics permission was initially granted for the Swedish segment of the study by the Uppsala Regional Ethics Committee on August 21, 2019 (chair Håkan Julius) and by the Swedish Ethical Review Authority for the extended study on May 27, 2022 (chair Louise Conradi). Each participating centre outside of Sweden obtained ethical clearance from their respective local authorities according to local regulations. In several countries, the study was categorised as an audit and neither formal ethics application nor informed consent was necessary. The study was registered at ClinicalTrials (NCT05519969) and was conducted in accordance with the Helsinki declaration and its subsequent revisions. Reporting follows the STROBE statement.^7^ Further information about the study is available at www.uu.se/eurofast.

Hospitals were eligible for participating if they had a minimum case load of 1000 paediatric cases annually. All anaesthetics administered during the study period were monitored for incidents of regurgitation, vomiting, or signs of pulmonary aspiration. Exclusion criteria were age ≥16 yr and procedures not requiring anaesthesia care. Data collection started at three sites in Sweden 2020, whereas centres in other countries joined after ethics approval and data transfer agreements were signed in 2022–3. Inclusion stopped at all sites on December 31, 2023.

For the denominator data, the number of procedures in children aged <16 yr was collected from each hospital's administrative system. Each site was also asked to state which preoperative fasting regimen they implemented during the study period. For the numerator data, when an aspiration event occurred the anaesthesia team filled in a comprehensive case report form (CRF) capturing background data, clinical course, and treatments. The CRF also included basic information about airway management and actual fasting times for solids, breast milk, and clear fluids. Three cohorts were identified based on each site's fasting regimen for clear fluids: (1) clear fluids allowed until the patient is called to theatre (a fasting regimen previously called 6-4-0 but in recent publications often ‘sip-til-send’ which more accurately indicates the practice of allowing ingestion of clear fluids until the patient is called to the operating theatre); (2) clear fluids encouraged until 1 h before induction (the ≥1 h group, based on the recent ESAIC guideline); and (3) clear fluids stopped at least 2 h before induction of anaesthesia (control group, in previous publications often called 6-4-2, indicating a 6-h limit for ingestion of solid food, 4 h for breast milk or infant formula, and 2 h for clear fluids). The resulting numerator and denominator data described above were used for calculating the incidence of aspiration.

The primary outcome was confirmed pulmonary aspiration defined as regurgitation or vomiting (in the text and tables simply referred to as regurgitation) of gastric contents leading to respiratory symptoms that require high-dependency unit or ICU admission and gastric content observed in the trachea or radiologic findings typical for aspiration pneumonia. As secondary outcomes, four categories of events were analysed: (1) regurgitation event with transient symptoms; (2) regurgitation event that leads to escalation of care such as unplanned admission for inpatient care, supplemental oxygen beyond the emergence phase, or treatment with antibiotics; (3) regurgitation event that leads to unplanned need for mechanical ventilation in the ICU; and (4) pulmonary aspiration that leads to death.^1^

### Statistical methods

Sample size calculation was based on an incidence of two confirmed pulmonary aspirations in 10 000 anaesthetics reported in a large multicentre audit in the UK, and a noninferiority limit of 4:10 000, resulting a minimum sample of 60 000 in each cohort for a power of 90% and an alpha of 0.025.^8^ The study plan included the option to stop inclusion when at least one liberal fasting cohort exceeded 60 000. Data were expressed as absolute numbers (incidence) or mean values (sd). Differences between cohorts were expressed as 95% confidence intervals (CIs) calculated using Mietinen–Nurmineńs method for confidence intervals of proportions.^9^ All statistical analyses were performed using R version 4.3.0. Noninferiority 95% CIs were thus calculated for the primary outcome of confirmed pulmonary aspiration and for transient aspiration and a composite outcome of escalation of care including patients admitted to the ICU without a confirmed aspiration diagnosis, often referred to as suspected pulmonary aspiration.^4^^,^^10^ For the secondary analysis of real clear fluid fasting times and the incidence of regurgitation events, a χ^2^ test was used.

## Results

**Fig 1:**
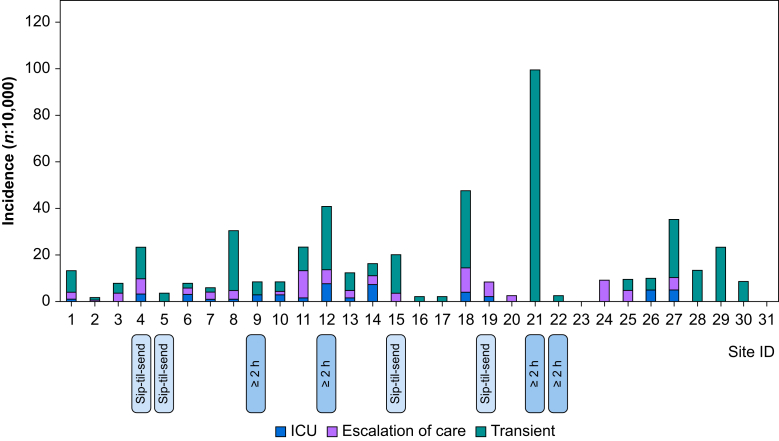
Incidence of regurgitation events categorised as transient (green), escalation of care (purple), and requiring postoperative intensive care (blue). The *x*-axis displays the 31 sites in order according to number of anaesthetics (largest to smallest). Each site's fasting regimen is indicated as sip-til-send (STS), ≥1 h clear fluid fasting (blank), or ≥2 h clear fluid fasting.

In total, 31 centres participated in the study (see Appendix 1) contributing a total of 306 900 anaesthetic procedures monitored for regurgitation events. The sip-til-send cohort included 34 028 anaesthetics, the ≥1 h cohort 251 021 anaesthetics, and the control cohort 21 851 anaesthetics. After exclusion of 17 cases that were patients >15 yr old, there were 420 registered regurgitation events including 286 categorised as transient (68%), 94 leading to escalation of care (22%), and 40 leading to postoperative intensive care (9.5%). There were no reported deaths. Figure 1 displays the incidences of the three categories at each participating centre. The total incidence (all categories) ranged from 0 to 41:10 000 (mean 9:10 000). The mean age of affected children was 6.5 (0–15) yr and 39% of cases were female. Patient characteristics and clinical features are summarised in Table 1.

**Table 1:** Patient characteristic and clinical data for the 420 regurgitation cases.

	Sip-til-send	≥1 h	≥2 h	Total
Age (yr)	6.5 (0–14)	6.3 (0–15)	7.0 (0–15)	6.5 (0–15)
Weight (kg), mean (sd)	27.2 (22)	26.3 (18)	28.0 (17)	26.7 (18)
Female, *n* (%)	14 (34)	120 (40)	28 (36)	164 (39)
ASA physical status, *n* (%)				
1	26 (54)	66 (20)	55 (73)	141 (34)
2	8 (17)	111 (38)	14 (19)	133 (32)
3	14 (29)	117 (40)	6 (8)	137 (33)
4	0	8 (3)	0	8 (1.9)
Procedure, *n* (%)				
Elective	40 (83)	237 (80)	68 (91)	345 (82)
Urgent (within 24 h)	6 (13)	31 (10)	4 (5.3)	41 (9.8)
Emergency	2 (4.2)	29 (9.8)	3 (4.0)	34 (8.1)
Surgical	41 (87)	217 (73)	68 (92)	326 (78)
Nonsurgical	6 (13)	82 (27)	6 (8.1)	94 (22)
Location, *n* (%)				
Operating theatre	48 (100)	228 (77)	73 (97)	349 (83)
Remote location	0	69 (23)	2 (2.7)	71 (17)

**Fig 2:**
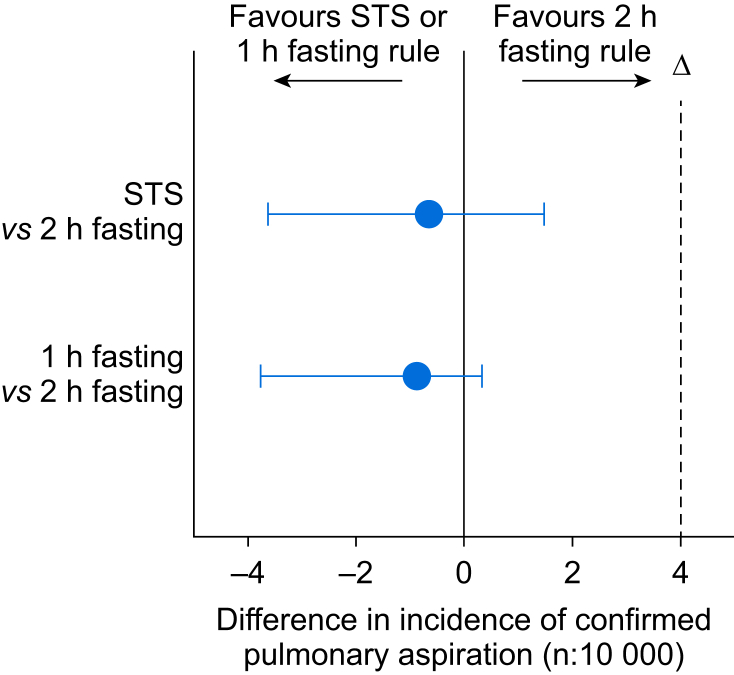
Number of events (incidence *n*:10 000) and 95% confidence intervals for the differences in confirmed pulmonary aspirations between sites with the ≥2 h preoperative clear fluid fasting rule (number of anaesthetics = 21 851) *vs* the ≥1 h rule (number of anaesthetics = 251 021) or the sip-til-send (STS) rule (number of anaesthetics = 34 028). D indicates the predefined noninferiority margin.

For the primary outcome of confirmed pulmonary aspiration, the incidence was 1.18:10 000 in the sip-til-send group, 0.96:10 000 in the ≥1 h group, and 1.83:10 000 in the ≥2 h control group. Both sip-til-send and ≥1 h clear fluid fasting regimens were statistically noninferior to ≥2 h clear fluid fasting with 95% CIs for the differences of –1.48 to 3.63 and –0.34 to 3.76, respectively (Table 2, Fig 2).

**Table 2:** Number of events (incidence *n*:10 000) and 95% confidence intervals (CIs) for the differences in incidence between centres with the ≥2 h preoperative clear fluid fasting rule *vs* the ≥1 h rule or the sip-til-send (STS) rule.

	Clear fluid fasting regimen				Differences (95% CI)	
	STS	≥1 h	≥2 h	Total	≥2 h *vs* ≥1 h	≥2 h *vs* STS
Number of centres	4	23	4	31		
Number of anaesthetics	34 028	251 021	21 851	306 900		
Number of events	41	302	77	420		
Categorised events: *n* (incidence)						
Transient	27 (7.9)	191 (7.6)	68 (31)	286 (9.3)	(16.8–31)	(15.8–31.9)
Escalation of care	10 (2.9)	780 (3.1)	4 (1.8)	94 (3.1)		
ICU	4 (1.2)	31 (1.2)	5 (2.3)	40 (1.3)		
Death	0	0	0	0		
Escalation of care + ICU	14 (4.1)	111 (4.4)	9 (4.1)	134 (4.4)	(–2.48 to 3.47)	(–3.42 to 4.06)
Confirmed pulmonary aspiration	4 (1.2)	24 (0.96)	4 (1.8)	32 (1.0)	(–0.34 to 3.76)	(–1.48 to 3.63)

The incidences of transient regurgitation events were 7.9:10 000 in the sip-til-send group, 7.6:10 000 in the ≥1 h clear fluid fasting group, and 31:10 000 in the ≥2 h clear fluid fasting control group. When compared with the standard ≥2 h clear fluid fasting regimen, both the sip-til-send and the ≥1 h clear fluid fasting regimens were noninferior as the incidence was significantly higher in the ≥2 h clear fluid fasting group in both cases (95% CI of the difference of 15.8–31.9 and 16.8–31.9 in 10 000, respectively). One centre using the ≥2 h clear fluid fasting regimen reported an exceptionally high rate of regurgitation with transient symptoms (41 events in 4124 anaesthetics). A sensitivity analysis performed by repeating the comparisons of transient regurgitation events without the latter outlier confirmed that both sip-til-send and ≥1 h clear fluid fasting were noninferior to ≥2 clear fluid fasting.

The incidence of aspiration events leading to postoperative escalation of care including intensive care was 4.1:10 000 in the sip-til-send group, 4.4:10 000 in the ≥1 h clear fluid fasting group, and 4.1:10 000 in the ≥ 2h clear fluid fasting group. Both the sip-til-send and the ≥1 h clear fluid fasting regimens were noninferior to the standard ≥2 h clear fluid fasting regimen regarding the incidence of serious aspiration events. The 95% CIs of the differences were –3.42 to 4.06 and –2.48 to 3.47 in 10 000, respectively.

The distribution of aspiration events and outcomes in children with recorded preoperative actual clear fluid fasting times <1 h, 1–2 h, or ≥2 h (regardless of group allocation according to fasting regimen) is displayed in Table 3. Aspiration events were recorded in 18 children with actual preoperative fasting times <1 h, resulting in transient symptoms in 12 cases and postoperative escalation of care in six cases, respectively. Amongst 54 children with aspiration and recorded fasting times between 1 and 2 h, transient symptoms were reported in 36 cases, escalation of care in 16 cases, and postoperative intensive care in two cases. In comparison, there were 233 children with actual fasting times ≥2 h; 147 transient aspirations, 58 leading to escalation of care and 26 needing intensive care. The distribution across the three categories was without statistical significance (*P*=0.24).

**Table 3:** Reported actual clear fluid fasting time in aspiration cases and their distribution amongst the categories of transient symptoms, escalation of care, ICU, and death. Mean (sd) values and absolute numbers (% of total number of events, *n*=303∗). ∗Missing actual fasting time data in 116 cases.

	Real clear fluid fasting time			
	<1 h	1–2 h	≥2 h	Total
Mean h (sd)	0.61 (0.24)	1.6 (0.29)	6.9 (4.7)	5.5 (4.7)
Range	0.25–0.95	1.0–1.95	2.0–21	0.25–21
*Categorised events*				
Transient	12 (3.9%)	36 (12%)	147 (48%)	195
Escalation of care	6 (2.0%)	16 (5.2%)	58 (17%)	80
ICU	0	2 (0.65%)	26 (8.5%)	28
Death	0	0	0	0
Total	18 (5.9%)	54 (18%)	231 (76%)	303

The CRF included fields for reporting pre-identified risk factors, displayed in Supplementary Table 3. Risk factors were found in 229 of 420 regurgitation events. Gastrointestinal pathology, whether anatomical or functional, was the most common risk factor for aspiration reported by the anaesthesia team (46% in total).

Hypoxaemia was the most common complication (33% of aspiration cases), followed by laryngospasm (15%), aspiration pneumonia (5.4%), and bronchospasm (4.4%) (Table 4). There were no cardiac arrests or aspirations leading to brain damage.

**Table 4:** Complications associated with the regurgitation events. Absolute numbers (% of the 410 cases with complications data).

Complication	n%
Hypoxaemia	134 (33)
Laryngospasm	61 (15)
Pneumonitis/pneumonia	22 (5.4)
Bronchospasm	18 (4.4)
Bradycardia	7 (1.7)
Airway obstruction by aspirate	3 (0.73)
Cardiac arrest	0
Brain damage	0
Other	3 (0.73)

Actual fasting times regarding solids, breast milk, and clear fluids for regurgitation cases are displayed in Supplementary Table 1. Among cases with intraoperative regurgitation, eight children had ingested solids within 6 h of anaesthesia induction, two were breast-fed <3 h before induction, and a total of 72 had clear fluids within 2 h of induction. Information regarding management of regurgitation and aspiration events can be found in Supplementary Table 2, and recorded risk factors for aspiration in Supplementary Table 3.

## Discussion

We conducted an adequately powered, pragmatic, prospective noninferiority study including patients from 31 hospitals from four continents of three different preoperative fasting regimens for clear fluids. The results demonstrate noninferiority for both sip-til-send and ≥1 h clear fluid fasting regarding confirmed pulmonary aspiration and regurgitation with transient symptoms, escalation of care, and aspiration requiring postoperative intensive care compared with standard 6-4-2 fasting.

The incidence of aspiration has decreased in the last three decades although differences in definitions and design make direct comparisons difficult.^1^ A number of observational studies have compared the incidence of pulmonary aspiration between children in centres with liberal clear fluid fasting regimens and those in centres with the standard 6-4-2 fasting rules. However, these results have limitations because of inadequate sample sizes to compare the incidence of aspiration. In an audit conducted in 11 Swiss hospitals with paediatric anaesthesia, including 22 766 children, the authors reported an incidence of confirmed aspiration (0.02%), suspected aspiration (0.09%), regurgitations (0.15%), and vomiting (0.37%).^10^ These numbers are comparable with the present study with rates of confirmed aspiration (0.01%), escalation of care (0.03%), and transient regurgitation events (0.08%). In the Swiss study, an inclusion criterium was implementation of the ≥1 h clear fluid fasting rule. In a similar audit involving 12 093 children from 15 hospitals in Germany and the Netherlands with four centres implementing 6-4-0 (sip-til-send), nine with the 6-4-1, and two using the 6-4-2 regimen, confirmed aspiration was reported in four cases (0.03%), with 10 cases of suspected aspiration (0.09%) and 31 cases of transient regurgitation (0.26%).^11^

The transition to liberal clear fluid fasting in children is already happening in Europe and elsewhere, facilitated by the recent ESAIC guideline which includes a strong recommendation to encourage intake of clear fluids up until 1 h before anaesthesia.^3^ However, the risks and benefits of replacing the (8-)6-4-2 regimen (8 h for a large meal, 6 h for a light meal, 4 h for breast milk, and 2 h for clear fluids) have been debated.^12^ The latter regimen has been applied widely since the first ASA guidelines were published in 1999.^2^ The main pragmatic argument for a change to more liberal fasting rules is that several audits have shown that prolonged fasting times often in excess of 8 h are common when applying the 6-4-2 regimen.^13^ In comparison, the German aspiration audit (NiKs) reported median (IQR) fasting times of 1.8 (0.9–3.8) h in sip-til-send centres and 2.5 (1.6–5.1) h in ≥1 h centres.^11^ Similarly, in the Swiss audit the median real fasting time was 2.6 (1.7–5.3) h.^10^ Quality improvement projects have shown that changing to ≥1 h from ≥2 h clear fluid fasting reduces real fasting times by >50%.^5^^,^^6^ The main physiologic arguments for short fasting times are that clear fluids empty from the stomach rapidly, and that dehydration, hypoglycaemia, and hypotension can be prevented, especially in vulnerable populations.^14^, ^15^, ^16^, ^17^

Our study did not collect denominator data regarding fasting times as the main focus was the effect of implemented fasting regimens on the incidence of aspiration rather than compliance to fasting rules. Although the latter query could have been interesting to study in such a large cohort, we chose to keep data collection as simple as possible to be able to enrol as many sites as possible. In the data from regurgitation cases, it is noteworthy that only 18 children with <1 h real clear fluid recorded fasting time were involved in any kind of aspiration event (Table 3). Among aspiration events requiring postoperative intensive care, 26 had fasted ≥2 h, two had fasted 1–2 h, and none had fasted <1 h for clear fluids. Furthermore, very few breaches of the fasting rules for solids or breast milk were evident in aspiration cases (Supplementary Table 1). This suggests that other factors than actual preoperative fasting times are likely causes of pulmonary aspiration. The incidence of confirmed pulmonary aspiration has decreased over the last three decades in spite of the introduction of more liberal fasting regimens, possibly because of increased awareness after publications such as the Fourth National Audit Project (NAP4) leading to improved airway management practices.^1^^,^^18^

The arguments against changing to liberal fasting regimens can be summarised in terms of futility and safety. Firstly, those who challenge the change argue that any child will tolerate 2 h of fasting and the change to ≤1 h neither makes a difference in physiological terms nor for the wellbeing of the child.^12^ We would argue that this confuses nominal fasting times (2 h *vs* ≤1 h) with real-world fasting times that are consistently much longer.^13^^,^^19^^,^^20^

Secondly, concerns have been raised about the safety of liberal fasting regimens. Although previous audits did not suggest increased rates of aspiration when liberal fasting regimens were applied, adequately powered noninferiority studies were lacking. We believe the present study fills this gap. A future meta-analysis of recent audits would provide even stronger evidence for the safety of reducing clear fluid fasting times. It would be facilitated by the fact that the CRFs for incident reporting in the NiKs audit, the Swiss audit and the present study are similar in design.^10^^,^^11^

For many years, a reduction in real preoperative fasting times was repeatedly emphasised in the discussion that, with the right organisational structure, short real fasting times could be achieved even with the 2-h rule for clear fluids. What sounds simple is much more difficult to implement in real-world ward and operating theatre organisation.

Prevention of prolonged fasting times requires easy rules that allow children to drink without interfering with the flow of theatre lists that are invariably subject to changes owing to cancellations or unexpected delays as a result of complicated surgery or anaesthesia. The change to the ≥1 h rule seems to overcome these logistic problems as it results in shorter actual fasting times.^5^^,^^6^ However, the great strength of the ‘sip-til-send’ rule is its simplicity. This can lead to reliable short fasting times without keeping track of exactly when and where each child ingested clear fluids. The low incidence of aspiration in the present study supports a transition to either sip-til-send or the ESAIC recommendations. Hospitals considering changing their practice will have to make individual decisions regarding which of these two strategies is most suitable for them.

This study should be interpreted in the context of its limitations. Firstly, the observational design of the study limits definitive causal conclusions. However, an RCT of different fasting regimens with at least 120 000 participants would hardly be feasible because of the huge workload. In addition, an RCT would not reflect clinical practice as did the present cohort design. It is challenging enough to change or even maintain a fasting regimen in a large hospital. Secondly, the absence of complete denominator data regarding comorbidity and fasting times is a limitation. The latter kind of data could have been useful to identify and quantify risk factors. However, risk factor analysis was not the main focus of the present study and has been adequately addressed in previous studies. Thirdly, the study plan was to aim for cohorts of at least 60 000 anaesthetics each, but if not feasible within a reasonable time frame, it would suffice with >60 000 in at least one liberal fasting category. At study completion, this number was safely exceeded for the main comparison group that implemented the ESAIC guideline, but not for the other two groups including the control group. However, although a larger final sample size would have provided even narrower CIs, the noninferiority results with the current sample size remain statistically valid. Fourthly, we cannot exclude confounding owing to differences in anaesthesia practice between participating centres and reporting bias owing to clinicians and centres possibly having different thresholds for reporting (under-reporting or over-reporting). The latter is illustrated by the strikingly high incidence of transient events at one of the hospitals in the control cohort. However, the study was widely announced and paediatric hospitals with all three types of clear fluid fasting regimen were encouraged to participate. Furthermore, repeated reminders and study updates were sent to the local co-ordinators during the data collection period to decrease the risk of study fatigue and under-reporting. The large sample size and the inclusion of at least four sites for each fasting regimen might compensate for individual site differences. In addition, the sensitivity analysis with exclusion of the outlier site with a high aspiration incidence did not yield a different result. Fifthly, in the present study we only had access to actual fasting data for 306 of the children with aspiration events. The decision to collect detailed data only in cases with adverse events was pragmatic, to maximise the number of collaborating centres. The drawback is that we could not analyse the effect of actual fasting time on aspiration. Instead, we settled for comparing the rate of adverse events in centres with different fasting regimens, which in the end provides the most relevant information for patients, parents, hospitals, and healthcare practitioners.

## Conclusions

In this study of paediatric patients observing three different fasting regimes, we found that sip-til-send and ≥1 h clear fluid fasting were noninferior to ≥2 h clear fluid fasting with regards to the primary outcome of confirmed aspiration, and secondary outcomes of regurgitation with transient symptoms and escalation of care after aspiration. These results provide evidence for the safety of reducing the minimum recommended preoperative fasting time for clear fluids from 2 h to ≤1 h.

## Authors' contributions

Study design: PF, PS

Conception: PF

Co-ordinated data acquisition and prepared data for analysis: ARM

Accessed and verified the data: PF, ARM

Data acquisition: all authors

Data analysis: all authors

Funding acquisition: RF

Wrote the first draft of the manuscript: PF

Critical revision of the manuscript: all authors

Approval of the final version of the manuscript: all authors

## Funding

Departmental funding from Uppsala University Hospital; 10.13039/501100004359Swedish Research Council (Vetenskapsrådet 2014–02569 and Vetenskapsrådet 2014–07606 to RF).

## Declaration of interests

PF is an unpaid member and treasurer of the Executive Board of the European Society of Paediatric Anaesthesiology. PAS is an unpaid member of the Board of Directors of the Society for Pediatric Anesthesia. The other authors declare no conflict of interest.

## Footnotes

Appendix A Supplementary data to this article can be found online at https://doi.org/10.1016/j.bja.2025.03.031.
