# Supplementary material for: Impact of liberal preoperative clear fluid fasting regimens on the risk of pulmonary aspiration in children (EUROFAST): an international prospective cohort study
Source: Br J Anaesth. 2025 May 26;135(1):141–7. doi: 10.1016/j.bja.2025.03.031 (PMC12226750; doi:10.1016/j.bja.2025.03.031)
Supplement: Multimedia component 1 [file mmc1.docx]

**Supplementary Table 1.**  **Actual fasting times registered for 306* regurgitation/aspiration cases.**

**Panel 1**. Data reported as mean (SD), range and number of cases in each category.

|  |  | Solids | Infant formula | Breast milk | Clear fluids |
| --- | --- | --- | --- | --- | --- |
|  |  |  |  |  |  |
| Transient | Mean (SD) | 14 (5.9) | 11 (7.8) | 5.0 (3.0) | 5.5 (5.4) |
|  | Range (n) | 0 - 41 (229) | 2.0 - 41 (38) | 2.0 - 8.8 (5) | 0.25 - 38 (194) |
|  |  |  |  |  |  |
| Escalation of care | Mean (SD) | 15 (4.6) | 10 (4.9) | 7.2 | 6.8 (7.0) |
|  | Range (n) | 5.8 - 37 (81) | 3.8 - 21 (9) | 7.2 - 7.2 (1) | 0.25 - 34 (83) |
|  |  |  |  |  |  |
| ICU | Mean (SD) | 12 (5.0) | 6.8 (1.2) | 11 (0.71) | 7.5 (4.9) |
|  | Range (n) | 2.0 - 22 (29) | 5.9 - 7.7 (2) | 10 - 11 (2) | 1.5 - 16 (29) |
|  |  |  |  |  |  |
| Total | Mean (SD) | 14 (5.5) | 11 (7.2) | 6,7 (3.4) | 6.1 (5.9) |
|  | Range (n) | 0 - 41) | 2 - 41 | 2 (11) | 0.25 - 36 (306) |

*Missing values in 116 of the 420 cases

**Panel 2.** Number of cases who had ingested solids, infant formula, breast milk (BM) or clear fluids (CF) closer to induction than recommended in fasting guidelines.

|  |  |  |  |  |  |  |
| --- | --- | --- | --- | --- | --- | --- |
| \|  \|  \|  \|  \|  \|  \|  \| \| --- \| --- \| --- \| --- \| --- \| --- \| --- \| \| Breach of guideline: \| \| Solids < 6 h \| Formula < 4 h \| BM < 3 h \| CF < 2h, ≥1h \| CF < 1 h \| \|  \|  \|  \|  \|  \|  \|  \| \| Transient \|  \| 5 \| 1 \| 1 \| 36 \| 12 \| \|  \|  \|  \|  \|  \|  \|  \| \| Escalation of care \|  \| 0 \| 1 \| 1 \| 16 \| 6 \| \|  \|  \|  \|  \|  \|  \|  \| \| ICU \|  \| 3 \| 0 \| 0 \| 2 \| 0 \| \|  \|  \|  \|  \|  \|  \|  \| \| Total \|  \| 8 \| 2 \| 2 \| 54 \| 18 \| |  |  |  |  |  |  |
|  |  |  |  |  |  |  |
|  |  |  |  |  |  |  |
|  |  |  |  |  |  |  |
|  |  |  |  |  |  |  |
|  |  |  |  |  |  |  |
|  |  |  |  |  |  |  |
|  |  |  |  |  |  |  |
|  |  |  |  |  |  |  |
|  |  |  |  |  |  |  |
|  |  |  |  |  |  |  |

**Supplementary Table 2**

Management of regurgitation and aspiration events (% of the 417 cases after exclusion of 3 cases without data on management). Each case could be subjected to more than one treatment.

| **Treatment** | **Sip-til-send** | **≥ 1h fasting** | **≥ 2h fasting** | **Total** | **%** |
| --- | --- | --- | --- | --- | --- |
|  |  |  |  |  |  |
| Bronchotracheal suctioning | 13 | 103 | 13 | 129 | 31% |
| Bronchodilator therapy | 5 | 26 | 5 | 36 | 9% |
| Diagnostic flexible bronchoscopy | 2 | 14 | 2 | 18 | 4% |
| Bronchoscopy with lavage | 1 | 8 | 1 | 10 | 2% |
| Rigid bronchoscopy | 0 | 0 | 0 | 0 | 0% |
| Chest radiograph | 6 | 48 | 7 | 61 | 26% |
| CPAP | 1 | 10 | 1 | 12 | 3% |
| ECMO | 0 | 0 | 0 | 0 | 0% |
| Unplanned O_2_ supplementation 2 h postop. | 6 | 22 | 6 | 34 | 8% |
| Unplanned FiO_2_ > 0.4 intraoperatively | 10 | 31 | 10 | 51 | 12% |
| Unplanned intraoperative intubation | 7 | 62 | 7 | 76 | 18% |
| Postoperative intubation | 1 | 14 | 1 | 16 | 4% |
| Unplanned postop ICU | 6 | 17 | 6 | 29 | 7% |
| Unplanned admission of outpatient | 5 | 23 | 5 | 33 | 8% |
| Antiobiotic therapy | 2 | 20 | 2 | 24 | 6% |
| Other | 3 | 16 | 3 | 22 | 5% |
| No treatment needed | 29 | 146 | 29 | 204 | 49% |

**Supplementary Table 3.** Risk factors. Absolute numbers (% of the 420 cases)

Gastrointestinal anatomy pathology 63 (28%)

Gastrointestinal mobility disorder 41 (18%)

Gastro-oesophageal reflux 33 (15%)

Neurological disease 27 (7%)

Light anaesthesia 22 (10%)

Urgent surgery 41 (9.7%)

Emergency surgery 34 (8.0%)

Opioid administration 15 (7%)

Difficult airway 11 (5%)

Lithotomy position 8 (3%)

Preoperative intensive care 7 (3%)
