# Supplementary material for: Impact of liberal preoperative clear fluid fasting regimens on the risk of pulmonary aspiration in children (EUROFAST): an international prospective cohort study
Source: Br J Anaesth. 2025 May 26;135(1):141–7. doi: 10.1016/j.bja.2025.03.031 (PMC12226750; doi:10.1016/j.bja.2025.03.031)
Supplement: Multimedia component 2 [file mmc2.docx]

**EUROFAST Collaborative Group*** (Helen Ahmad^1^, Victoria Barlow^2^, Rebecca Binks^3^, Stephanie Bowler^4^, Lynn Fenner^5^, Gil Gavel^6^, James Gaynor^7^, Rowan Hardy^8^, Tova Hannegård Hamrin^9^, Helena Hansson^10^, Baptiste Hengy^11^, Xantha Holmwood^5^, Francesca Holt^12^, James Ip^13^, Jimmy José^12^, Rumiko King^14^, Jānis Kolbergs^15^, Zuzana Kusnirikova^16^, Peter Larsson^9^, Li Yen Lieu^6^, Prashanth Reddy^17^,

Mari Roberts^18^, David Rosen^19^, Nada Sabourdin^20^, Takpal Sandhu^21^, Sanjiv Sharma^4^, John Shubhaker^1^, Adam Shuttleworth^8^, Karina Sifontes Romero^21^, Amrit Singh^6^, Deborshi Sinha^3,^ Rachel Smith^22^, Gabriel Soares de Sousa^23,24^, Ricardo Vieira Carlos^24^, Vanessa Rigterink^25^, Shamil Tanna^1^, Julia Taylor^26^, Christopher Todd^4^, Carolina Tormo de las Hera^21^, Ahmed Uslu^27^, Joris Vunderlinckx^28^, Stephanie Wallis^18^, Toni Weber^29^ Gun Wiberg^10^).

1. Moorfield's Eye Hospital, London, UK
2. Royal Manchester Children’s Hospital, Manchester University NHS Foundation Trust, UK
3. Nottingham Children's Hospital, Nottingham, UK
4. East Lancashire Hospitals NHS Trust, UK
5. Salisbury District Hospital, Wiltshire, UK
6. Royal Aberdeen Children's Hospital, Aberdeen, UK
7. Norfolk and Norwich University Hospital, Norwich, UK
8. Royal United Hospitals Bath NHS Foundation Trust, Bath, UK
9. Department of Physiology and Pharmacology, Karolinska Institutet, Solna, Sweden
10. Falun District Hospital, Falun, Sweden
11. Hospices Civils de Lyon, Femme Mère Enfant Hospital, University of Lyon, Université Claude Bernard Lyon 1, France
12. Children's Health Ireland, Crumlin, Dublin, Ireland
13. Great Ormond Street Hospital, London, UK
14. St. George’s Hospital, London, UK
15. Children's Clinical University Hospital, Riga, Latvia
16. Royal Hospital for Children's, Glasgow, UK
17. Royal Stoke University Hospital, Stoke-on-Trent, UK
18. Children’s Hospital for Wales, Cardiff, UK
19. Children’s Hospital of Ottawa, University of Ottawa, Canada
20. Assistance Publique – Hôpitaux de Paris, Paris, France
21. University Hospital Niño Jesús, Madrid, Spain
22. Royal Bolton Hospital, Bolton, UK
23. Hospital Infantil Menino Jesus, São Paulo, Brasil
24. Hospital das Clínicas HCFMUSP, Faculdade de Medicina, Universidade de São Paulo, São Paulo, Brazil
25. Hannover Medical School, Hannover, Germany.
26. Royal National Orthopaedic Hospital, Stanmore, UK
27. Baskent University Ankara Hospital, Ankara, Turkiye
28. Ziekenhuis Oost-Limburg, Genk, Belgium
29. Children´s Hospital in Sankt Augustin, Sankt Augustin, Germany

*Children´s Hospital of Philadelphia and Uppsala University Hospital are not listed here since there were no collaborative authors, only main authors from these two centres.
